# Human Breast Cancer Cells Demonstrate Electrical Excitability

**DOI:** 10.3389/fnins.2020.00404

**Published:** 2020-04-30

**Authors:** Mafalda Ribeiro, Aya Elghajiji, Scott P. Fraser, Zoë D. Burke, David Tosh, Mustafa B. A. Djamgoz, Paulo R. F. Rocha

**Affiliations:** ^1^Department of Electronic and Electrical Engineering, Centre for Biosensors, Bioelectronics and Biodevices (C3Bio), University of Bath, Bath, United Kingdom; ^2^Department of Biology and Biochemistry, Centre for Regenerative Medicine, University of Bath, Bath, United Kingdom; ^3^Neuroscience Solutions to Cancer Research Group, Department of Life Sciences, Imperial College of London, London, United Kingdom

**Keywords:** breast cancer, metastasis, voltage-gated sodium channels, multi-electrode arrays, bioelectronics, sensors, electrophysiology

## Abstract

Breast cancer is one of the most prevalent types of cancers worldwide and yet, its pathophysiology is poorly understood. Single-cell electrophysiological studies have provided evidence that membrane depolarization is implicated in the proliferation and metastasis of breast cancer. However, metastatic breast cancer cells are highly dynamic microscopic systems with complexities beyond a single-cell level. There is an urgent need for electrophysiological studies and technologies capable of decoding the intercellular signaling pathways and networks that control proliferation and metastasis, particularly at a population level. Hence, we present for the first time non-invasive *in vitro* electrical recordings of strongly metastatic MDA-MB-231 and weakly/non-metastatic MCF-7 breast cancer cell lines. To accomplish this, we fabricated an ultra-low noise sensor that exploits large-area electrodes, of 2 mm^2^, which maximizes the double-layer capacitance and concomitant detection sensitivity. We show that the current recorded after adherence of the cells is dominated by the opening of voltage-gated sodium channels (VGSCs), confirmed by application of the highly specific inhibitor, tetrodotoxin (TTX). The electrical activity of MDA-MB-231 cells surpasses that of the MCF-7 cells, suggesting a link between the cells’ bioelectricity and invasiveness. We also recorded an activity pattern with characteristics similar to that of Random Telegraph Signal (RTS) noise. RTS patterns were less frequent than the asynchronous VGSC signals. The RTS noise power spectral density showed a Lorentzian shape, which revealed the presence of a low-frequency signal across MDA-MB-231 cell populations with propagation speeds of the same order as those reported for intercellular Ca^2+^ waves. Our recording platform paves the way for real-time investigations of the bioelectricity of cancer cells, their ionic/pharmacological properties and relationship to metastatic potential.

## Introduction

Cancer is one of the major killers of humans with many problems remaining in its clinical management, from primary diagnosis to treatment of advanced disease. Breast cancer (BCa) is the most common type of cancer and leading cause of cancer death in women with over 2 million new cases reported in 2018 (Bray et al., 2018). BCa-related deaths often occur through dysregulated tumor signaling compounded by metastasis (Sever and Brugge, 2015). A strongly metastatic and aggressive form of BCa is the triple-negative breast cancer (TNBC), where genes for the estrogen receptor (ER) and progesterone receptor (PR) are not expressed, and there is no amplification of the human epidermal growth factor receptor 2 (HER2) (Perou et al., 2000; Lee and Djamgoz, 2018). This renders TNBC particularly difficult to treat as the commonly applied hormone therapies rely on targeting at least one of these three receptors (Al-Mahmood et al., 2018). The MDA-MB-231 cell line is used commonly as a model of TNBC and has a mesenchymal phenotype (Hero et al., 2019). Its underlying intracellular signaling pathways have been studied using both genomic analyses and expression profiling (Perou et al., 2000; Sorlie et al., 2001; Neve et al., 2006). Indeed, these cells exhibit *in vitro* characteristics representative of cancer cell behavior *in vivo* (Sever and Brugge, 2015).

In recent years, aberrant expression of ion channels and transporters (ICTs) has been implicated in various stages of the cancer process, such as cell proliferation, apoptosis, migration, and invasiveness (Lastraioli et al., 2015). This highlights the importance of studying cancer cell electrophysiology. In particular, voltage-gated sodium channels (VGSCs) may play a potentiating role in metastasis (Arcangeli et al., 2008; Fraser and Pardo, 2008; Prevarskaya et al., 2010; Cuddapah and Sontheimer, 2011; Djamgoz et al., 2014). VGSCs are typically known for their role in producing action potentials in excitable cells. Djamgoz advanced the view (“Celex Hypothesis”) that it is the *de novo* VGSC expression and the resulting “membrane excitability” that would promote cancer cell aggressiveness and, ultimately, metastasis (Djamgoz, 2011; Djamgoz et al., 2014). Such studies have been aided by the availability of rich pharmacology for ion channels, as well as genetic studies. In human and rodent cell lines, single-cell patch clamp recordings revealed that VGSCs can be blocked with tetrodotoxin (TTX) (Grimes et al., 1995; Roger et al., 2003). In turn, TTX application would lead to suppression of invasiveness *in vitro* and metastasis *in vivo* (Grimes et al., 1995; Roger et al., 2003; Fraser et al., 2005; Yildirim et al., 2012; Djamgoz et al., 2019).

Single-cell patch clamp recording is used for investigating membrane currents or voltages associated with ion channels upon the application of an external stimulus, under current or voltage clamp conditions, respectively. This sort of intracellular monitoring under an applied electrical stimulus can provide detailed information at the cellular level. However, for reliable *in vitro* models and studies of phenomena such as cancer cell invasion, the application of an external stimulus is likely to influence natural interlinked cell-cell dynamics. Additionally, cancer cell proliferation, invasion and metastasis typically involve a cell cohort, and hence it would be advantageous to monitor the network phenomena together with the electrical activity of single cells. Changes in the cell cohort during metastasis, when cancer cells spread to tissues and organs beyond the original tumor’s location, are of paramount clinical importance. Hence, patch clamping alone lacks the ability for non-invasive network mapping within the tumor bulk. Monitoring unbiased cell invasion at a population level coupled with individual cellular measurements is therefore critical for understanding tumor proliferation, invasion and metastasis.

The technical challenge is that the membrane currents associated with VGSC activity in cancer cells possess amplitudes which are orders of magnitude smaller compared to neurons. Commercially available electrophysiology systems such as multi-electrode arrays (MEAs) comprise a high density array of electrodes that are either planar or three-dimensional, with a diameter ranging between 10 and 100 μm typically embedded on top of an insulating MEA substrate (Spira and Hai, 2013). This set-up allows for neuronal cells to adhere and their electrical activity readily recorded and modulated. However, the size of the electrodes contributes toward debilitatingly high impedances and the monitoring window is set in the kHz range where neuronal firing typically occurs. Low-frequency events are therefore filtered out. To overcome these technical challenges, we devised a sensitive detection method based on large electrodes, with areas in the order of mm^2^. Our low-impedance electrodes make use of a large Helmholtz-Gouy-Chapman double-layer capacitance. The measured current signal is enhanced by the high sealing resistance between the confluent cell layer and the Au electrode, and by a gain factor proportional to the large electrode capacitance allowing for small-amplitude signals to be detected (Medeiros et al., 2016; Rocha et al., 2016a, b; Cabello et al., 2019).

In this paper, we show that the strongly metastatic BCa cells can be electrically monitored using the proposed sensitive electrode system. The recordings from MDA-MB-231 cells reveal the presence of asynchronous spikes with magnitudes ranging from 40 to 140 pA. The electrical activity of MDA-MB-231 cells significantly surpasses the attenuated electrical activity of the weakly metastatic cell line, MCF-7. By using the pharmacological inhibitor TTX, we show that the asynchronous spikes are primarily caused by the opening and closing of the VGSCs, in direct agreement with VGSC expression occurring specifically in BCa cells of strong metastatic potential (Fraser et al., 2005). In addition, we note the sporadic occurrence of “Random Telegraph Signal” (RTS) noise. RTS patterns were less frequent than the asynchronous VGSC signals. Characterization of the RTS noise revealed the presence of a low-frequency signal across the cell cohort with propagation speeds of the same order as those reported for intercellular “Ca^2+^ waves” (Rizaner et al., 2016). Our findings suggest that the electrical activity recorded from the MDA-MB-231 cells is a metastatic phenomenon. Our recording system enables the real time investigation of the bioelectricity of BCa cells and corroborant cohort signaling.

## Materials and Methods

### Maintenance of Breast Cancer Cell Lines

The MDA-MB-231 and MCF-7 cells were cultured in Dulbecco’s Modified Eagle Medium (DMEM; Sigma) containing 5% v/v fetal bovine serum (FBS; Gibco) and 4 mM L-Glutamine (Sigma). The cells were harvested once a confluence of 70% or over was achieved. The cells were washed with phosphate buffered saline (PBS; Sigma) and dissociated using 0.05% Trypsin-EDTA solution (Sigma) at 37°C for 3 min. A cell density of 1 × 10^6^ cells/mL was deposited on the device for each experiment. Images were acquired using either an M205 Leica stereo microscope (cells on electrodes) or a Leica DMIRB microscope fitted with a Nikon DXM1200C digital camera.

Trypan blue exclusion assay was used to determine viability of cells plated on MEAs compared to the tissue culture dish (Fraser et al., 2005). Briefly, a MEA was placed in a 35 mm tissue culture dish (Falcon) and pre-treated for 20 min with 0.01 mg/mL poly-L-lysine (Sigma) to promote cell adhesion. Cells were plated at a density of 1 × 10^6^ cells/mL and allowed to attach overnight. The media was then replaced with 0.1% trypan blue solution. Following incubation for 10 min at 37^*o*^C, the trypan blue solution was replaced with 1 mL fresh cell culture medium and the cells were viewed at 100x magnification under a dissection microscope (Vickers Instruments). The percentage of dead cells was determined from 30 randomly selected fields of view for either the MEA or the dish. This procedure was repeated in 4 independent experiments.

### MEA Design and Manufacturing

The MEA device consisted of a silicon dioxide substrate onto which four pairs of circular electrodes were deposited, each with an area of 2 mm^2^, with an inter-electrode distance of 1.8 mm. This was achieved by evaporating a 10 nm layer of chromium followed by a 50 nm layer of gold through a shadow mask with the desired electrode design. An Edwards Auto 360 thermal evaporator was used. The electrodes were connected to measurement pads at the edges of the device through strip lines of negligible area. In order to contain the culture medium and cells over the electrodes, a previously manufactured poly(methyl methacrylate) (PMMA) well was secured onto the MEA before plating the cells.

The MEA was then further enclosed in an autoclaved container and the electrodes covered in 0.01 mg/mL poly-l-lysine (Sigma) to promote cell adhesion. This was applied and left for 5 min, washed three times with MilliQ water and left to stand for 1.5 h prior to depositing the cells. Measurements started at least 1 h after the cell seeding. The cell segmentation plugin within ImageJ was used to quantify the number of cells on the electrodes which amounted between 4,000 and 5,000 cells. The container with the seeded MEA was kept in an incubator (Midi 40; Thermo Scientific) at 37°C and 5% CO_2_ and connected to low-noise cables to perform the electrical measurements. The current between the 2 mm^2^ electrode pair was measured using a low-noise current amplifier (SR570; Stanford Research) with an amplification factor of 10 nA/V. This data was then plotted using a dynamic signal analyser (35670A; Agilent) and in-house LabVIEW software connected via a high speed GPIB interface. The instrumentation was further contained within a Faraday cage and connected with low-noise cables to minimize external interference.

### Pharmacology

To determine the origin of the electrical activity and following prior research relating to the role of VGSCs in metastasis (Fraser et al., 2005; Chioni et al., 2009), a highly specific VGSC inhibitor, TTX (Alomone Labs), was applied to the cells in culture. Three experiments were conducted using 20 μM, following the reported value of TTX toxicity in earlier studies (Fraser et al., 2005). For these experiments, 2 μL of 10 mM TTX was combined with 20 μL of the culture medium on the MEA and the mixture reintroduced into the system, obtain final concentrations of 20 μM of TTX in 1 mL volume on the MEA. This was left for an hour and the recording of the electrical current of the cell cohort was continued. Finally, the MEA was washed thrice with fresh medium at 37°C. The recovery of the ensuing electrical behavior was seen shortly after washing the TTX out and monitored over an additional 24 h.

Additionally, to determine the origin of the RTS signals, the cells were incubated with 1 mM ethylene gylcol-bis(β-aminoethyl ether)-N,N,N′,N′-tetraacetic acid (EGTA; Sigma) while their electrical activity was being monitored. Following a 10 min treatment, the EGTA medium was discarded, the cells were washed thrice with normal medium and the medium was finally replaced with fresh normal medium. The electrical monitoring resumed for another 24 h.

## Results and Discussion

### Detection Method and Sensor Viability

**FIGURE 1:**
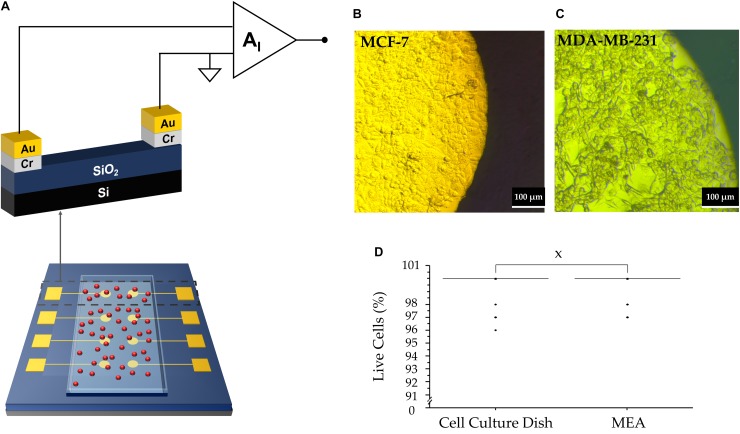
**(A)** Representative diagram of the MEA device with a layer of adherent cells (depicted in red). **(B)** MC7 cells forming a confluent layer on electrode. The bright part denotes cells on top of the Au electrode; the darker region indicates the cells outside the Au electrode. **(C)** MDA-MB-231 cells attached to the electrode. **(D)** Box plot of cell viability studies indicating no significant statistical difference between viability of cells on the MEA vs. culture dish (“X” represents *P* = 0.98).

Figure 1A shows the design and materials chosen for the MEA devices which were used to perform the extracellular measurements. The sensor comprises two circular electrodes, with one of the electrodes acting as a measuring electrode and the other serving as a counter electrode. The interface between the cells and the electrodes can be described using an equivalent circuit model. When an electrolyte is in close contact with an electrode, a Helmholtz-Gouy-Chapman double layer is formed consisting of a resistance, *R*_*D*_ in parallel with a capacitance, *C*_*D*_. Cells deposited on the measuring electrode, generate a voltage, *v*_*S*_(t) The resultant signal loss is modeled by a spreading resistance, *R*_*C*_. A transimpedance amplifier was used in this setup to measure the corresponding displacement current, *i*_*S*_(*t*), as described earlier (Medeiros et al., 2016):

(1) $$i_{S}\left(t\right)=\frac{dv_{S}}{dt}\cdot C_{D}\left(1-e^{\left(-t/\tau \right)}\right)$$

where τ≅*R*_*C*_*S*_*D*_ and corresponds to the time constant for the charging and discharging of the network. *C*_*D*_ acts as a multiplication factor and is dependent on the electrode area. The spatial resolution is considerably reduced due to the large electrode area. The measured current signal is the sum of each individual cell’s contribution on the electrode surface. The signal recorded using our large electrode area is the sum of all active cell contributions. Uncorrelated cell activity appears as noise and low magnitude asynchronous spikes. Thus, we argue that the asynchronous activity is mostly due to uncorrelated single cell activity. The origin is discussed in the next section. We note that during the recordings, both MDA-MB-231 cells and MCF-7 cells were confluent, maintained their familiar mesenchymal phenotype and adhered well to the electrodes. Figures 1B,C are of MCF-7 and MDA-MB-231 cells, respectively, taken 24 h after cell seeding. Furthermore, their viability was not affected by contact with the electrode, as depicted in Figure 1D.

### Recording of Asynchronous Electrical Currents

**FIGURE 2:**
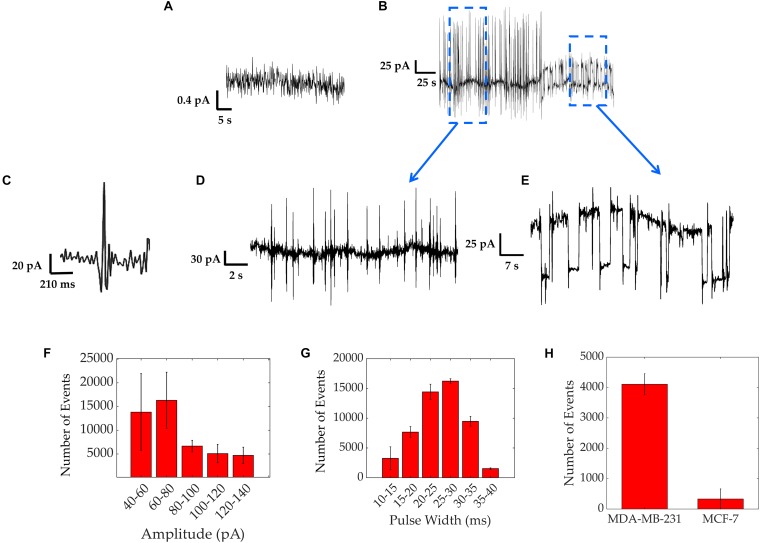
Electrical activity of MDA-MB-231 cells recorded over a period of up to 3 days. **(A)** Baseline current measured with only medium over the electrodes’ surface, showing a current of 0.5 pA. **(B)** Typical asynchronous spiking activity. **(C)** An expanded trace showing a fast-asynchronous spike. **(D)** Detailed section of fast spiking activity. **(E)** Detailed section of square shaped pulses. **(F,G)** Results from spike characterization over a 12 h period of asynchronous spiking activity of MDA-MB-231 cells, showing the distribution of amplitudes with bin intervals of 20 pA each **(F)** and pulse widths with bin intervals of 5 ms each **(G)** Bars represent average values of all 3 repeats and error bars represent standard deviation. **(H)** Experiment results comparing the spike counts obtained from 24 h measurements on strongly metastatic MDA-MB-231 cells vs. weakly metastatic MCF-7 cells, showing a significantly smaller spike count for the latter.

The bioelectricity of the MDA-MB-231 cell populations was recorded over time, for up to 72 h. All electrical recordings were repeated in three independent experiments. Prior to introducing the cells, the baseline current seen using only cell culture medium on gold (Au) electrodes coated with Poly-L-Lysine was about 0.5 pA, as shown by Figure 2A and in agreement with previous work (Rocha et al., 2016b).

Figure 2B shows the two typical spiking patterns recorded from MDA-MB-231 cells. The first electrical pattern consists of asynchronous bipolar spikes. A high magnification asynchronous spike is depicted in Figure 2C and a group of these bipolar spikes is shown in Figure 2D. The average spike magnitude ranged from 40 to 140 pA, with most events falling between 60 and 80 pA, as seen in Figure 2F. To further quantify the recorded asynchronous pattern, we analyzed the data from three different experiments and extracted the distribution of asynchronous spike widths (Figure 2G). The distribution follows a Gaussian curve with a peak at around 30 ms, in good agreement with the timescales reported using patch clamp recordings on MDA-MB-231 cells (Fraser et al., 2005). We acknowledge the rare presence of spike magnitudes reaching up to 300 pA with spike widths of a few seconds, in agreement with previous investigations where longer amplitudes result from the activation of larger ensembles of cells (Rocha et al., 2016a, b, 2018). These were less frequent as depicted in the event count of Figure 2F. As a control, the electrical activity of MCF-7 cells was also measured in the same conditions as the MDA-MB-231 cells. MCF-7 cells are known to be weakly/non-metastatic and do not express functional VGSCs (Rizaner et al., 2016) and hence should exhibit reduced electrical activity in comparison to MDA-MB-231 cells. The electrical activity for both cell lines was compared as a spike count over the course of 24 h and is shown in Figure 2H. The spike count comparison, over three different experiments, confirms that MDA-MB-231 cells are 90% more electrically active than the MCF-7 cells. The MCF-7 cells sporadically exhibited bipolar spikes. These results are consistent with the electrogenicity of cancer associated with cells of strong metastatic potential (Fraser et al., 2005; Yildirim et al., 2012; Driffort et al., 2014; Nelson et al., 2015).

### Effect of TTX

**FIGURE 3:**
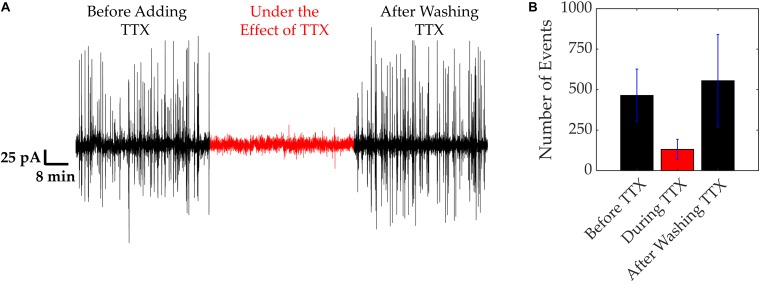
VGSC activity of MDA-MB-231 cells. VGSC activity was blocked using TTX (20 μM). **(A)** Current trace showing electrical activity before, during and after application of TTX. **(B)** Quantification of the spikes recorded in **(A)**. Number of spikes were measured in time bins of 6 min. Bars represent average values of all 3 repeats and error bars represent SDs.

In order to demonstrate the dependence of electrical activity on the presence of functional VGSCs, cells were treated with the neurotoxin TTX (Figure 3). Under normal culture conditions, cells were electrically active exhibiting asynchronous spikes with amplitudes of 60–150 pA (Figure 3). Following the addition of TTX this electrical activity decreased, with currents reaching up to 20 pA, as depicted in Figure 3A. Upon the removal of TTX, the electrical activity of the MDA-MB-231 cells resumed, as highlighted and quantified with spike counts in Figures 3A,B. The time bins in Figure 3B correspond to 6 min portions before, during, and after TTX. Before the TTX was added, an average of 460 ± 180 spikes were observed. This count reduced to an average of 130 ± 60 after 1–2 min while TTX was present. After the TTX was washed out, the number of spikes increased to 550 ± 280, and the original asynchronous spikes returned.

These results demonstrate a link between the fast, electrical “spiking” of the MDA-MB-231 cells and VGSC activity. TTX, a well-known VGSC blocker, reduced the extracellular electrical activity of these cells. Upon washout of the TTX, the cells resumed their previous electrical activity, confirming the rapid reversibility of the TTX action and the lack of any toxicity from the TTX concentration used. These results agree with our earlier study showing that intra-tumoral injection of TTX suppressed metastasis in a rat model of prostate cancer (Yildirim et al., 2012). Also, more recently, Yang et al. (2020) showed that lowering extracellular sodium does significantly hyperpolarize the membrane potential.

### Random Telegraph Signal Noise

RTS noise can be characterized in terms of its “up” and “down” time. The “up” time refers to the duration of time when the square pulse is above the baseline level. The “down” time refers to the duration of time when the square pulse has returned to and remains at the baseline level. Two-level RTS is defined as a Lorentzian spectrum in the frequency domain (Machlup, 1954). The Lorentzian spectrum gives the frequency above which the spectrum rolls-off as f^–2^, conditioning the harmonic mean of both times, “up” and “down.” When the RTS amplitude is sufficiently higher than the background noise, the Lorentzian spectrum can be used to estimate both time constants (Kolhatkar et al., 2003). A theoretical current PSD can also be derived from a two level signal as follows (Machlup, 1954):

(2) $$\frac{1}{\tau_{eff}}=\frac{1}{\tau_{UP}}+\frac{1}{\tau_{DOWN}}$$

(3) $$S_{I}\left(\omega \right)=4\left(\delta I^{2}\right)\left(\frac{\tau_{eff}}{\tau_{UP}+\tau_{DOWN}}\cdot \frac{\tau_{eff}}{1+\omega^{2}\tau_{eff}^{2}}\right)$$

Where ω = 2π*f* is the angular frequency and ∂⁡*I* is the amplitude of the current pulses.

**FIGURE 4:**
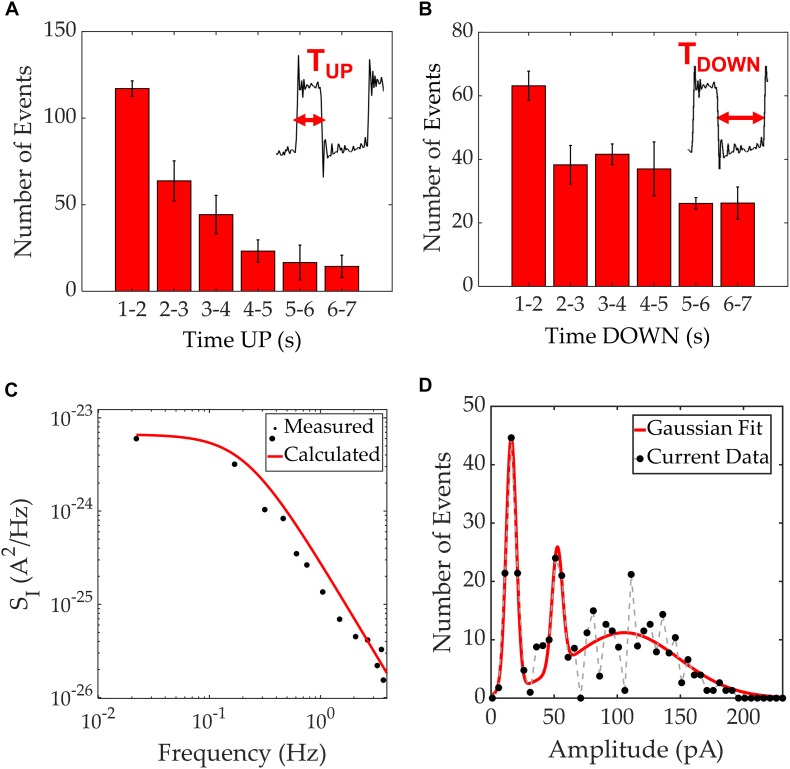
Time up/down characterization for square pulses seen in MDA-MB-231 current recordings. **(A)** Distribution of time “up” values with the inset showing the boundaries for this measurement. **(B)** Distribution of time “down” values with the inset showing the boundaries for this measurement. **(C)** Current PSD of the square pulses extracted from one of the experiments (black scatter plot) and a calculated PSD using the time constants τ*_*UP*_* = 1.1 s and τ*_*DOWN*_* = 2.5 s. A good agreement between theory (curve) and experimental data (points) is apparent. **(D)** Amplitude histogram of the current magnitudes recorded with a resolution of 5 pA, agglomerating 1260 RTS events which occurred during 3 different experiments. In red, a Gaussian fit guides the eye, to 3 distinct peaks.

The second electrical pattern revealed a wide square pulse shape as depicted in Figures 2B,E. This pattern consists of square pulses that maintain their amplitude between two different and well-defined states (inset of Figure 4A). The square-like pulses were characterized in terms of the length of time they are at an “up” state, τ*_*UP*_*, vs. a “down” state, τ*_*DOWN*_*. Since spikes with pulse widths in the order of <1 s are typically associated with previously seen VGSC activity, this analysis focused on pulses lasting 1 s or more.

The characterization of RTS is shown in Figures 4A,B with the time at an “up” state being typically 1–3 s, and the time at an “down” state around 1–5 s. Occasionally, these time intervals reached durations of 5 seconds or more. Figure 4C illustrates the PSD for the RTS noise, which shows the expected Lorentzian spectrum. The Lorentzian spectrum was calculated using (Equation 3) and is represented as a solid black line. Using the values τ*_*UP*_* = 1.1 s and τ*_*DOWN*_* = 2.5 s, one can model the RTS data and extract the overall characteristic times within 1260 RTS events. The experimental data is plotted in black dots illustrating a good match between the analytical model and the experimental data. In addition, the histogram of the recorded RTS amplitudes reveals three gaussian peaks at three distinct amplitude levels; 16, 52, and 107 pA and full width at half maximums of 9.6, 12.9, and 90.5 pA, respectively (Figure 4D). The amplitude histogram comprised 1260 RTS events extracted from 3 independent experiments. Patch clamp recordings have previously revealed the presence of RTS patterns on single-channel currents (Neher and Sakmann, 1976). The presence of RTS has been referred to stochastic processes of ion channels. However, in our work, as opposed to measuring a single cell, we record a cell cohort. We argue that the stochastic nature of individual ion channels combine within the surrounding cell cohort to create a transient behavior on long time-scales (Coombes et al., 2004), that could lead to a localized extracellular traveling wave. Considering the electrode area and duration of each pulse, it is possible to estimate the wave speed. When a wave enters the measurement electrode, the potential increases relative to the counter electrode, resulting in an induced displacement current through the double layer capacitance. This appears as an increase in current in the electrical measurements and once the wave has left the measurement electrode, the current decreases back to its original level (Rocha et al., 2016a; Cabello et al., 2019). The “up” states shown in the inset of Figure 4A, which go from the start of a pulse to when it returns to baseline level, typically range between 1 and 3 s. These timings were obtained when performing measurements on the 2 mm^2^ electrodes, which have a diameter of 1.6 mm. Therefore, the resultant wave speeds were of the order of hundreds of micrometers per second. These results are in close agreement with reported values for intercellular Ca^2+^ waves (Kuga et al., 2011).

**FIGURE 5:**
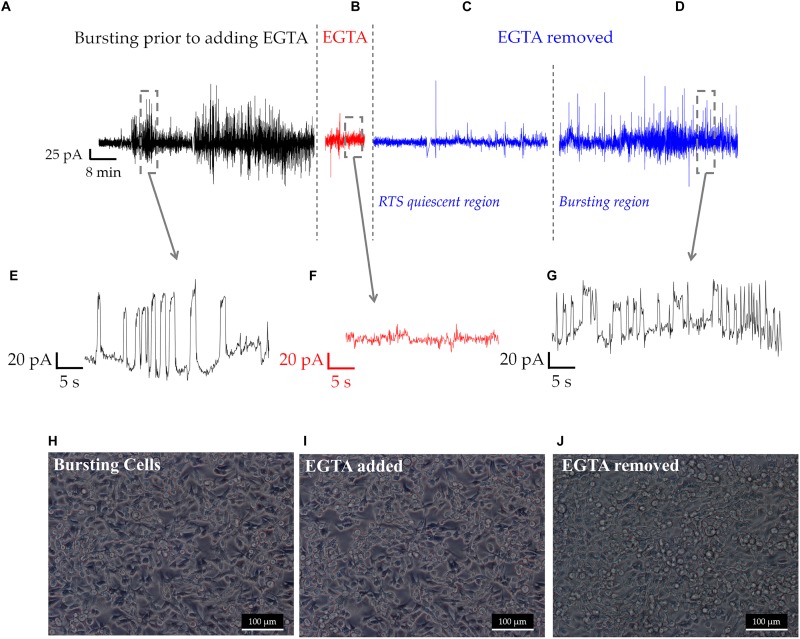
An overview of a long-term recording of a population of MDA-MB231 cells, at different experimental stages **(A–D)**. **(A)** RTS and asynchronous bursting prior to adding the chelating agent EGTA. **(B)** Effect of EGTA on the RTS activity during the 10 min treatment. **(C)** RTS quiescent region, where some occasional, low amplitude asynchronous spikes and some RTS activity resumes. **(D)** Recovery of RTS activity seen 8 h following EGTA wash. **(E–G)** Time traces of the different regions prior, during and following EGTA treatment. **(H–J)** Show the morphological changes to MDA-MB-231 cells throughout EGTA experiments, with tight junctions being clearly visible in **(H)**, decreased in **(I)** and forming again in **(J)**.

To determine whether cell-cell calcium signaling was responsible for RTS patterns, the extracellular chelating agent EGTA was added to cultures for 10 min. Under normal conditions, cells exhibited RTS signals with amplitudes between 20 and 100 pA, as seen in Figures 5A,E. In addition, we also observed asynchronous spiking activity, similar to the one depicted in Figure 2D. In the presence of 1 mM EGTA, RTS signals were abolished. Yet, some asynchronous oscillations reaching up to 20 pA were observed (Figures 5B,F). Following EGTA removal and the addition of fresh normal medium, the cells remained quiescent for several hours before resuming RTS signals with amplitudes of 20–100 pA (Figures 5C,D,G). It is important to note that removal of calcium from cell culture medium disrupts the tight junctions between the cells which might explain the decrease in cell electrogenicity, during the first few hours following EGTA removal (Mestre et al., 2017). Our analysis of cell morphology before during and after adding EGTA is depicted in Figures 5H–J, respectively. The tight junctions started to re-establish once EGTA is washed and fresh normal media is reintroduced (Figure 5J; Rothen-Rutishauser et al., 2002).

Calcium signaling is well known to orchestrate important events such as cell migration and metastasis (White, 2017). For calcium to regulate migration, localized and temporally complex signals, within cell domains exist (White, 2017). The generation and maintenance of long lasting calcium signals, in the seconds order, have been reported (Saint et al., 1992; Crill, 1996) in such domains, and linked with invasiveness in breast and colon cancer (House et al., 2010; Rizaner et al., 2016). The RTS analysis, ultimately, could unfold these multifactorial and tightly regulated communication within cell cohorts and extracellular space. Thus, more experiments need to be undertaken to unambiguously understand the origin of RTS and to link RTS to collaborative calcium transients.

## Conclusion and Future Perspective

This work revealed, for the first time, the extracellular electrical activity produced by MDA-MB-231 and MCF-7 cells, which was recorded using highly sensitive MEAs comprising gold electrodes with an area of 2 mm^2^. It was found that the strongly metastatic MDA-MB-231 cells are electrically active with spontaneous spiking, whilst the MCF-7 cells showed only very low-level electrical excitability. This is consistent with MDA-MB-231 cells being electrically excitable in line with their metastatic ability (Djamgoz, 2011; Djamgoz et al., 2014). The electrical behavior of MDA-MB-231 cells reveal an asynchronous pattern identified primarily as the product of their VGSC activity, as demonstrated by the TTX experiments. A slower signal pattern was also occasionally observed with similar characteristics to that of RTS noise. The inhibitory action of EGTA and the calculated wave speed for the RTS pattern suggests the involvement of intercellular Ca^2+^ waves. Ion channels involved in cell migration would be expected to regulate intracellular Ca^2+^ signaling, most likely coupling extracellular stimuli to cell migration. Therefore, this work demonstrated the possibility of using non-invasive, highly sensitive transducer devices to examine and pinpoint the origin of the electrical behavior of cancer cells. In future, it may be possible to use the sensor platform to investigate the pharmacological properties and action of drugs on cancer cell ensembles in a clinical setting.

## Data Availability Statement

The datasets generated and/or analyzed during the current study are available from the corresponding author on reasonable request.

## Author Contributions

PR and MD: conceptualization, resources, project administration, and funding acquisition. MR and AE: methodology, writing – original draft preparation. MR, SF, and AE: investigation. MR: data curation. PR, AE, and MD: writing – review and editing. PR, MD, DT, and ZB: supervision.

## Conflict of Interest

The authors declare that the research was conducted in the absence of any commercial or financial relationships that could be construed as a potential conflict of interest.
